# Supplementary material for: Reduction in Placental Metal and Metalloid in Preeclampsia: A Case–Control Study
Source: Nutrients. 2024 Mar 7;16(6):769. doi: 10.3390/nu16060769 (PMC10974158; doi:10.3390/nu16060769)
Supplement: Supplementary file 1 [file nutrients-16-00769-s001.zip › nutrients-2862480-supplementary.pdf]

nutrients

Supplementary Materials

Reduction in Placental Metal and Metalloid in Preeclampsia: A Case–Control Study

Yanhui Hao [1,†], Wen Yu [1,†], Jiaying Wu [2], Yingyu Yue [3], Yanting Wu [1], Hefeng Huang [1,*] and Weibin Wu [2,*]

1 Obstetrics and Gynecology Hospital, Fudan University, Shanghai 200011, China; yhao13@fudan.edu.cn (Y.H.); 17301050270@fudan.edu.cn (W.Y.); yanting_wu@163.com (Y.W.)
2 The International Peace Maternity and Child Health Hospital, School of Medicine, Shanghai Jiao Tong University, Shanghai 200030, China; wujiaying0570@163.com
3 Shanghai Medical College, Fudan University, Shanghai 200031, China; 22301050201@m.fudan.edu.cn
* Correspondence: huanghefg@hotmail.com (H.H.); wuweibin01@sjtu.edu.cn (W.W.)
† These authors contributed equally to this work.

Catalogue

Table S1. Median (IQR) concentrations of placental metal and metalloid measured in preeclampsia subgroups by FGR. 3
Table S2. Median (IQR) concentrations of placental metal and metalloid measured in healthy control and preeclampsia subgroups by gestational age.......... 3
Figure S1. Placental Ca distribution across gestational age (weeks) in the healthy control group and the preeclampsia (PE) group, categorized by maternal pre-pregnancy body mass index (BMI) groups. BMI as weight in kilograms divided by height in meters squared. Maternal BMI were categorized into three groups recommended by the Working Group on Obesity in China: BMI <18.5 was defined as Underweight, BMI≥24.0 kg/m^2 was defined as overweigh & Obesity.......... 4
Figure S2. Placental Cr distribution across gestational age (weeks) in the healthy control group and the preeclampsia (PE) group, categorized by maternal pre-pregnancy body mass index (BMI) groups. BMI as weight in kilograms divided by height in meters squared. Maternal BMI were categorized into three groups recommended by the Working Group on Obesity in China: BMI <18.5 was defined as Underweight, BMI≥24.0 kg/m^2 was defined as overweigh & Obesity.......... 5
Figure S3. Placental Mn distribution across gestational age (weeks) in the healthy control group and the preeclampsia (PE) group, categorized by maternal pre-pregnancy body mass index (BMI) groups. BMI as weight in kilograms divided by height in meters squared. Maternal BMI were categorized into three groups recommended by the Working Group on Obesity in China: BMI <18.5 was defined as Underweight, BMI≥24.0 kg/m^2 was defined as overweigh & Obesity.......... 6
Figure S4. Placental Fe distribution across gestational age (weeks) in the healthy control group and the preeclampsia (PE) group, categorized by maternal pre-pregnancy body mass index (BMI) groups. BMI as weight in kilograms divided by height in meters squared. Maternal BMI were categorized into three groups recommended by the Working Group on Obesity in China: BMI <18.5 was defined as Underweight, BMI≥24.0 kg/m^2 was defined as overweigh & Obesity.......... 7
Figure S5. Placental Co distribution across gestational age (weeks) in the healthy control group and the preeclampsia (PE) group, categorized by maternal pre-pregnancy body mass index (BMI) groups. BMI as weight in kilograms divided by height in meters squared. Maternal BMI were categorized into three groups recommended by the Working Group on Obesity in China: BMI <18.5 was defined as Underweight, BMI≥24.0 kg/m^2 was defined as overweigh & Obesity.......... 8
Figure S6. Placental Cu distribution across gestational age (weeks) in the healthy control group and the preeclampsia (PE) group, categorized by maternal pre-pregnancy body mass index (BMI) groups. BMI as weight in kilograms divided by height in meters squared. Maternal BMI were categorized into three groups recommended by the Working Group on Obesity in China: BMI <18.5 was defined as Underweight, BMI≥24.0 kg/m^2 was defined as overweigh & Obesity.......... 9
Figure S7. Placental Zn distribution across gestational age (weeks) in the healthy control group and the preeclampsia (PE) group, categorized by maternal pre-pregnancy body mass index (BMI) groups. BMI as weight in kilograms divided by height in meters squared.......... 10
Figure S8. Placental Se distribution across gestational age (weeks) in the healthy control group and the preeclampsia (PE) group, categorized by maternal pre-pregnancy body mass index (BMI) groups. BMI as weight in kilograms divided by height in meters squared.......... 11
Figure S9. Placental Cd distribution across gestational age (weeks) in the healthy control group and the preeclampsia (PE) group, categorized by maternal pre-pregnancy body mass index (BMI) groups. BMI as weight in kilograms divided by height in meters squared.......... 12
Figure S10. Placental As distribution across gestational age (weeks) in the healthy control group and the preeclampsia (PE) group,

categorized by maternal pre-pregnancy body mass index (BMI) groups... 13
Figure S11. The correlation of metals/metalloids measured in placental for the whole study population (n = 176) 14
Figure S12. The correlation of metals/metalloids measured in placental for the healthy control subjects (n = 113) 15
Figure S13. The correlation of metals/metalloids measured in placental for the preeclampsia group (n = 63) 16
Supplemented information on the assay of metal and metalloid in placenta................................. 16

Table S1. Median (IQR) concentrations of placental metal and metalloid measured in preeclampsia subgroups by FGR.

	Healthy control subjects n = 113	Preeclampsia without FGR n = 49	Preeclampsia with FGR n = 14	*p*-Value
**Magnesium**	1809.58 (1500.01,2340.72)	1496.13 (1199.6 , 1897.06)	1278.4 (923.33 , 1703.87)	**0.006**
**Calcium**	3341.57 (2208.47,6907.35)	2419.17 (1399.99,3369.36)	1648.74 (762.83,2363.44)	**0.002**
**Chromium**	18.65 (10.65,22.65)	9.59 (7.51 , 16.37)	8.42 (6.74 , 11.79)	**<.001**
**Manganese**	2.51 (1.7,3.09)	1.95 (1.35 , 2.51)	1.82 (1.15 , 2.43)	**0.005**
**Iron**	1554.66 (1239.73,1960.29)	1055.93 (883.22 , 1435.87)	806.1 (647.66 , 894.21)	**<.001**
Cobalt	4.83 (0.13,6.98)	4.26 (2.28 , 6.69)	4.21 (3.11 , 5.71)	0.898
**Copper**	13.69 (11.29,17.04)	11.37 (10.0 , 13.3)	10.77 (8.42 , 13.54)	**<.001**
**Zinc**	165.31 (141.18,199.22)	118.39 (98.69 , 142.76)	109.96 (76.45 , 128.68)	**<.001**
**Selenium**	3.21 (2.56,4.15)	2.47 (2.18 , 2.84)	2.25 (1.59 , 2.74)	**<.001**
**Cadmium**	0.24 (0.17,0.36)	0.22 (0.15 , 2.35)	0.20 (0.17 , 1.38)	0.827
**Arsenic**	0.33 (0.29,0.39)	0.23 (0.19 , 0.29)	0.25 (0.19 , 0.28)	**<.001**

Table S2. Median (IQR) concentrations of placental metal and metalloid measured in healthy control and preeclampsia subgroups by gestational age.

Metal and Metalloid	Healthy Control n = 113	Term Preeclampsia n = 33	Preterm Preeclampsia n = 20	Extremely Preterm Preeclampsia n = 10	*p*-Value
**Mg**	1809.58 (1500.01–2340.72)	1437.01 (1166.51–1757.01)	1645.19(1160.97,1846.6)	1472.48(1226.08,1820.85)	**<.001**
**Ca**	3341.57 (2208.47,6907.35)	2457.94 (1687.26,3598.42)	2006.478(762.834,2692.064)	2335.174(883.41,2990.359)	**0.004**
**Cr**	18.65 (10.65–22.65)	8.66 (7.22–13.45)	9.41(7.32,17.79)	12.6(8.46,19.08)	**<.001**
**Mn**	2.51 (1.70–3.09)	1.89 (1.16–2.31)	1.91(1.2,2.69)	2.36(1.86,2.69)	**0.012**
**Fe**	1554.66 (1239.73–1960.29)	1064.32 (894.21–1453.42)	923.98(789.85,1130.09)	826.86(706.09,1006.56)	**<.001**
Co	4.83 (0.13–6.98)	4.44 (2.28–6.69)	3.44(1.39,5.26)	5.12(2.93,5.76)	0.327
**Cu**	13.69 (11.29–17.04)	10.82 (9.53–13.51)	11.48(10.3,13.53)	10.15(9.88,12.74)	**0.019**
**Zn**	165.31 (141.18–199.22)	115.98 (92.23–136.54)	126.45(98.94,163.39)	123.07(106.05,138.17)	**<.001**
**Se**	3.21 (2.56–4.15)	2.46 (2.08–2.89)	2.45(2.13,2.82)	2.48(1.9,2.74)	**<.001**
Cd	0.24 (0.17–0.36)	0.20 (0.14–0.27)	0.21(0.19,0.95)	0.27(0.17,0.28)	0.952
**As**	0.33 (0.29–0.39)	0.22 (0.19–0.27)	0.26(0.2,0.3)	0.28(0.26,0.31)	**<.001**

Values are presented as Median (P25, P75), p value was based on the Wilcoxon-Mann-Whitney test * Preterm, delivery after 28 weeks of gestation and less than 37 weeks of gestation; Extremely preterm, delivery before 28 weeks of gestation. Values are presented as median (P25, P75), P values was based on the Wilcoxon-Mann-Whitney test for the comparison between subgroups of preeclampsia with or without extremely preterm. Abbreviation: As: arsenic; Ca: calcium; Cd: cadmium; Co: cobalt; Cr: chromium; Cu: copper; Fe: iron; Mg: magnesium; Mn: manganese; Se: selenium; Zn: zinc.

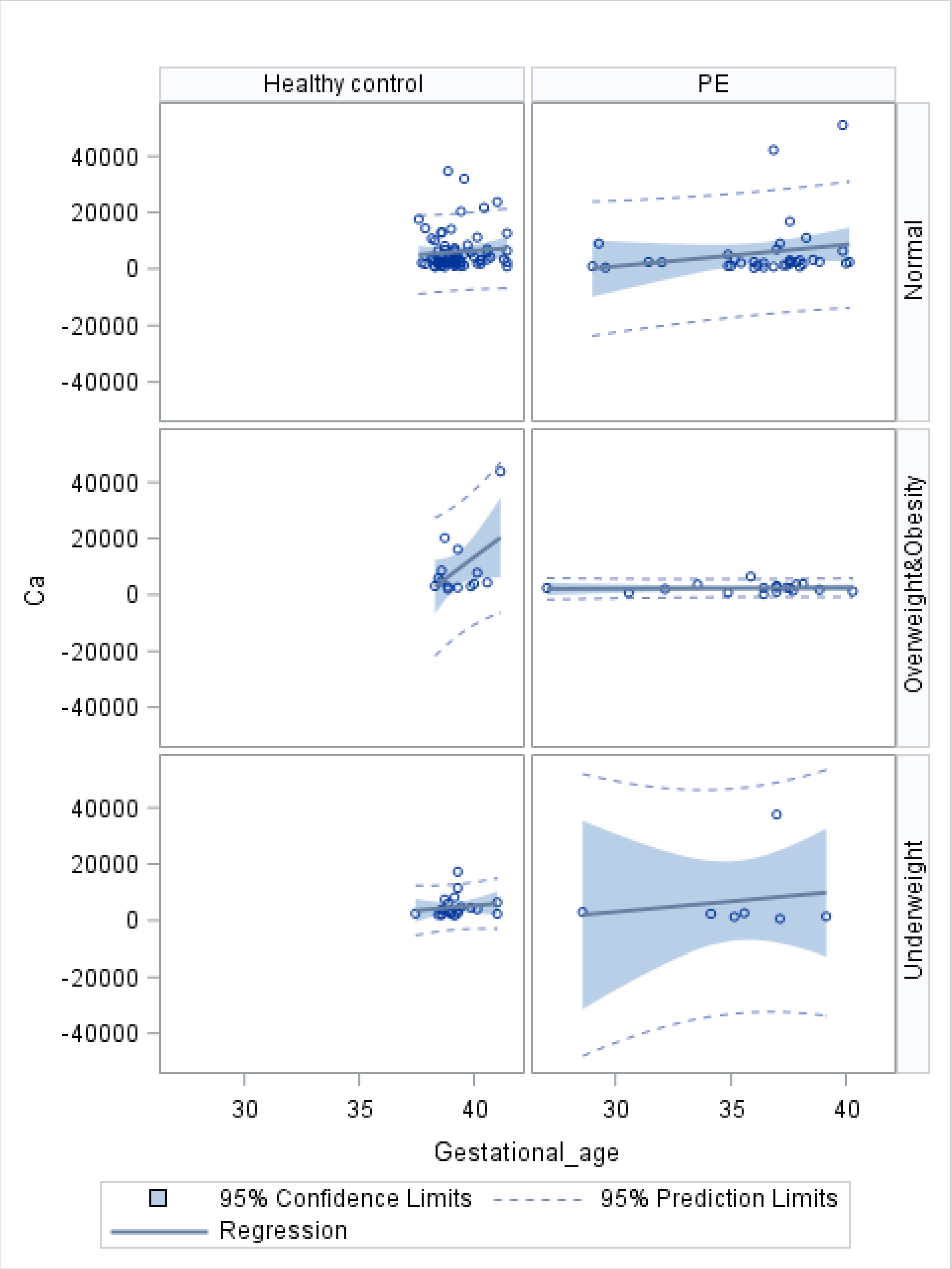

Figure S1. Placental Ca distribution across gestational age (weeks) in the healthy control group and the preeclampsia (PE) group, categorized by maternal pre-pregnancy body mass index (BMI) groups. BMI as weight in kilograms divided by height in meters squared.

Maternal BMI were categorized into three groups recommended by the Working Group on Obesity in China: BMI <18.5 was defined as Underweight, BMI≥24.0 kg/m^2 was defined as overweigh & Obesity.

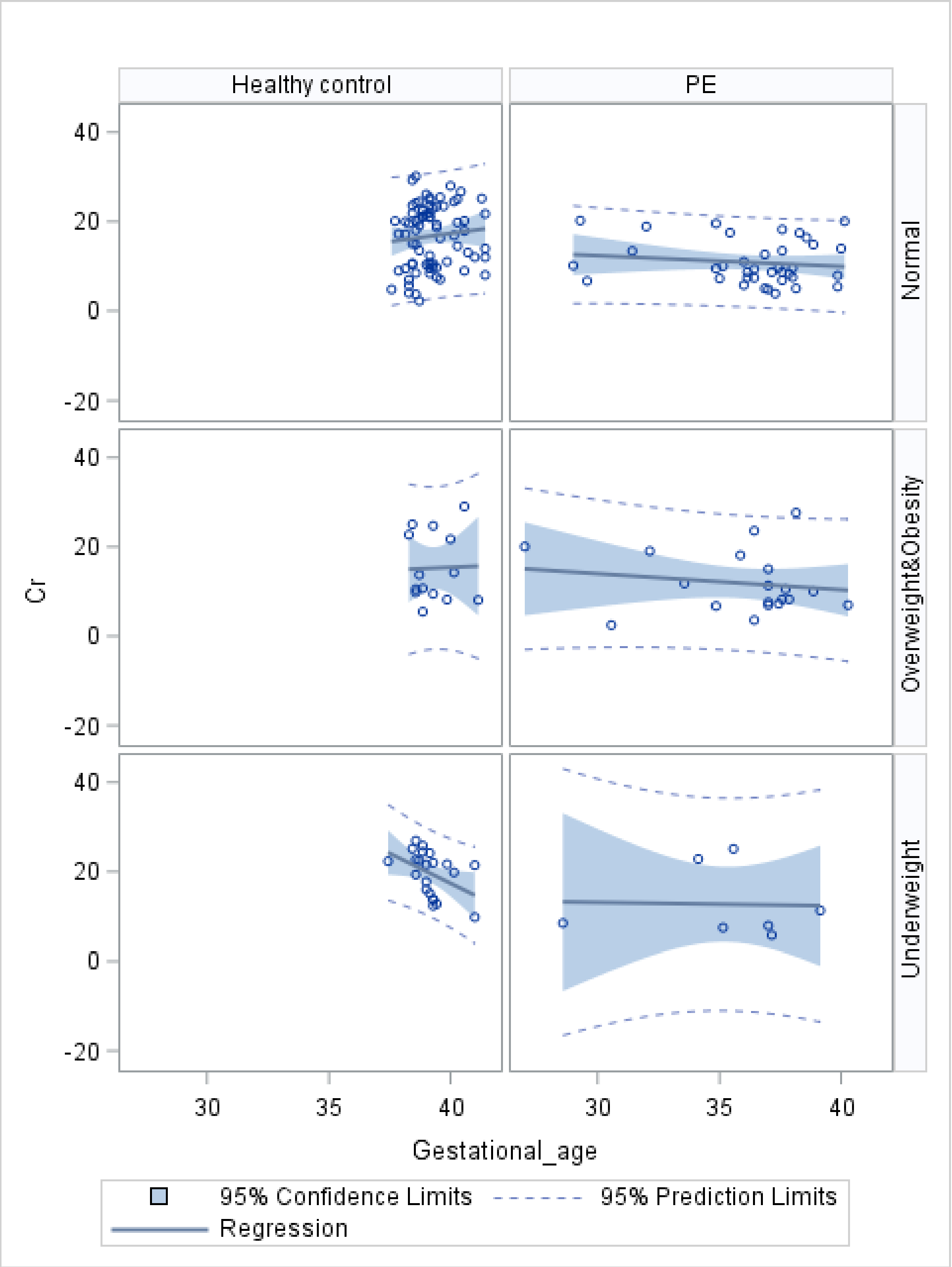

Figure S2. Placental Cr distribution across gestational age (weeks) in the healthy control group and the preeclampsia (PE) group, categorized by maternal pre-pregnancy body mass index (BMI) groups.

BMI as weight in kilograms divided by height in meters squared. Maternal BMI were categorized into three groups recommended by the Working Group on Obesity in China: BMI <18.5 was defined as Underweight, BMI≥24.0 kg/m^2 was defined as overweigh & Obesity.

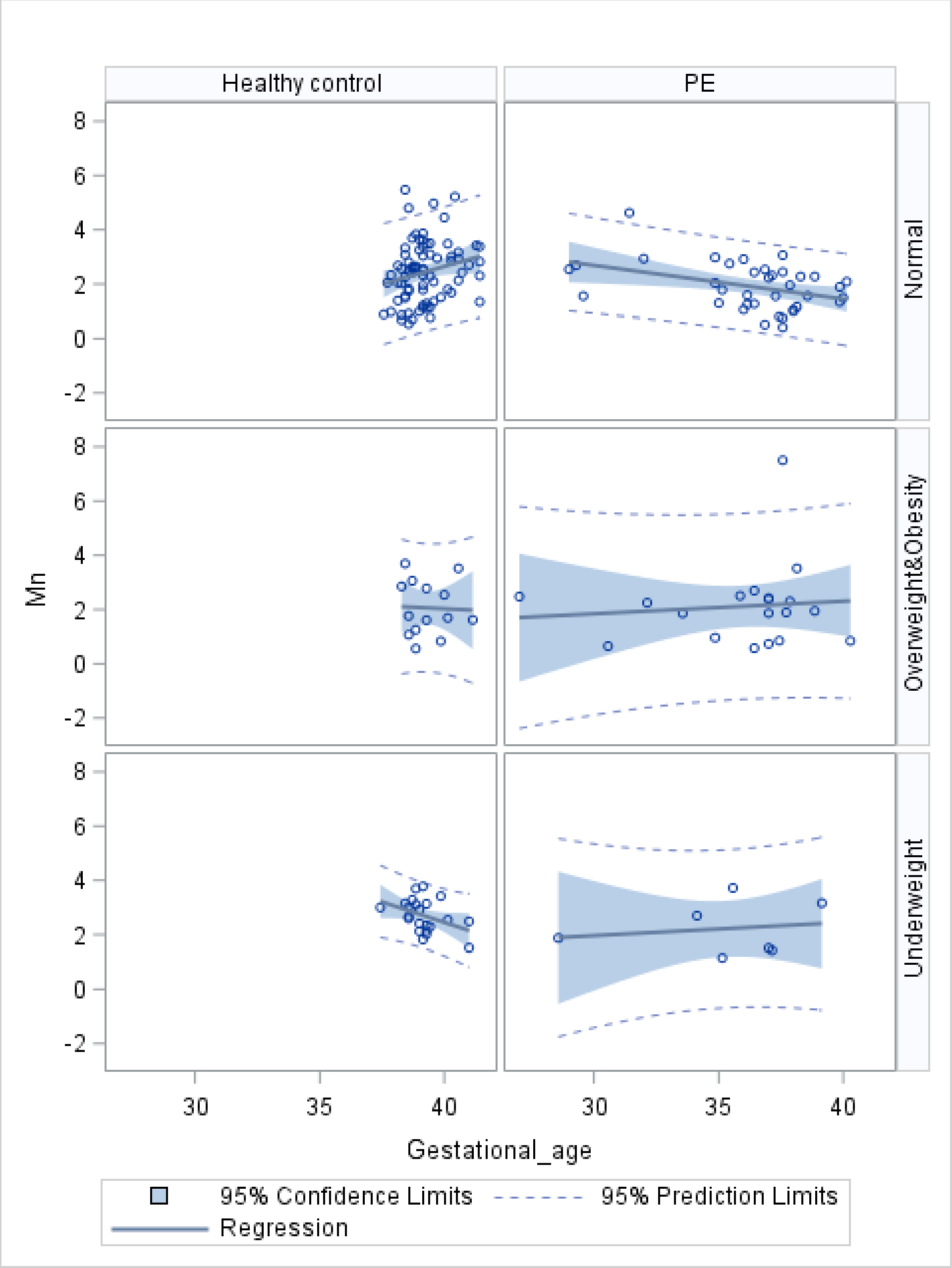

Figure S3. Placental Mn distribution across gestational age (weeks) in the healthy control group and the preeclampsia (PE) group, categorized by maternal pre-pregnancy body mass index (BMI) groups. BMI as weight in kilograms divided by height in meters squared.
Maternal BMI were categorized into three groups recommended by the Working Group on Obesity in China: BMI <18.5 was defined as Underweight, BMI≥24.0 kg/m² was defined as overweigh & Obesity.

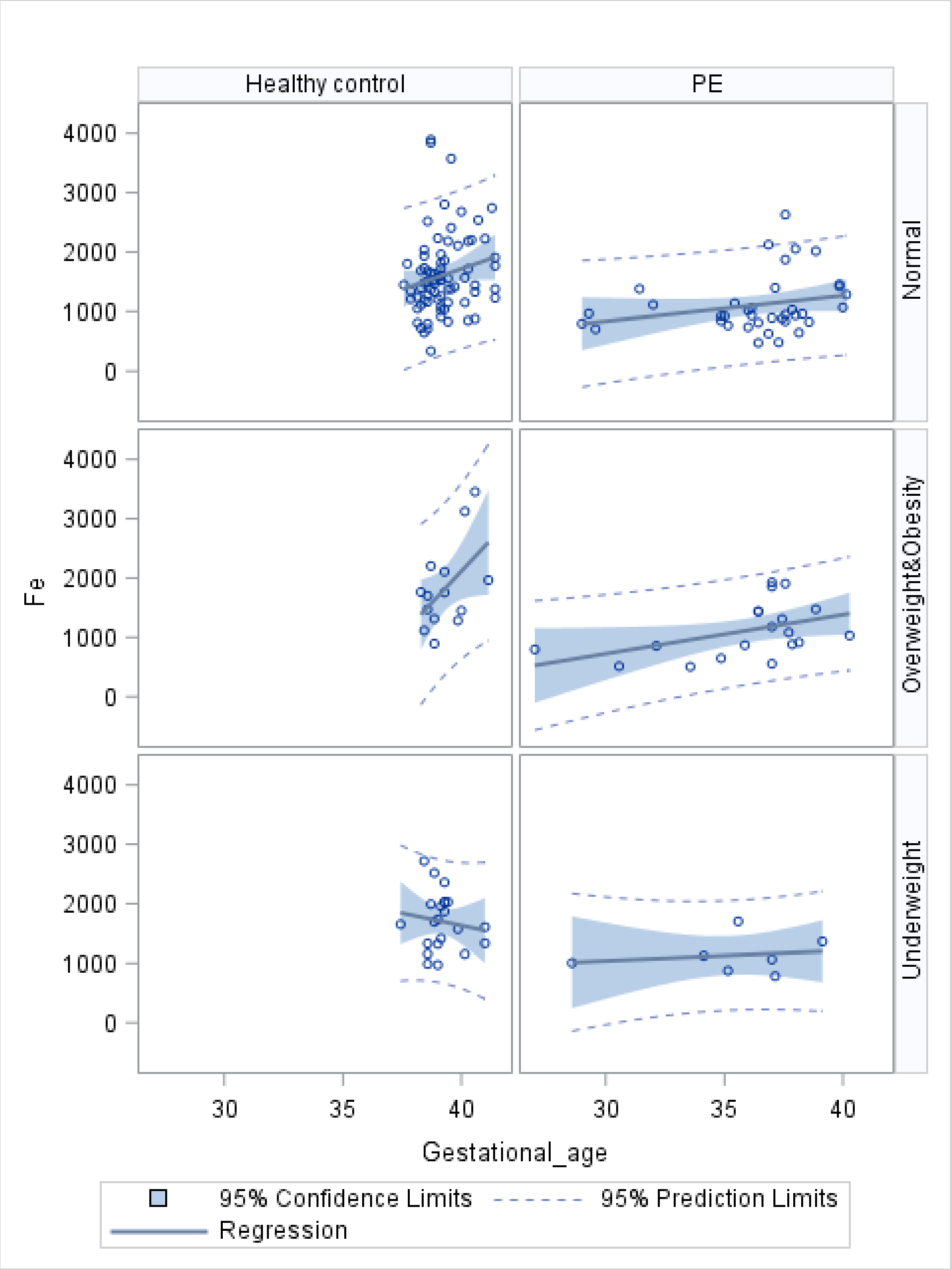

Figure S4. Placental Fe distribution across gestational age (weeks) in the healthy control group and the preeclampsia (PE) group, categorized by maternal pre-pregnancy body mass index (BMI) groups. BMI as weight in kilograms divided by height in meters squared.
Maternal BMI were categorized into three groups recommended by the Working Group on Obesity in China: BMI < 18.5 was defined as Underweight, BMI ≥ 24.0 kg/m^2 was defined as overweigh & Obesity.

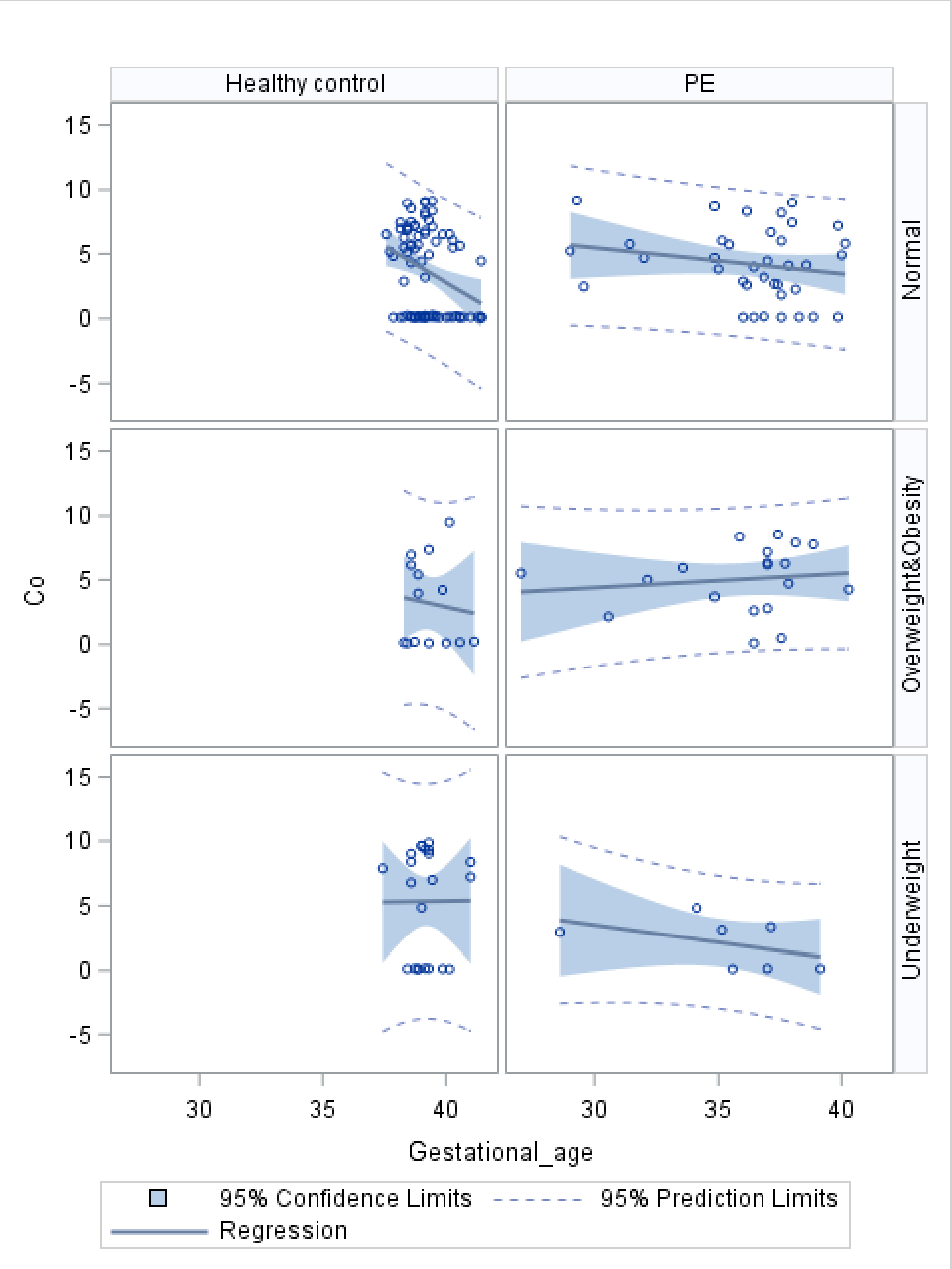

Figure S5. Placental Co distribution across gestational age (weeks) in the healthy control group and the preeclampsia (PE) group, categorized by maternal pre-pregnancy body mass index (BMI) groups. BMI as weight in kilograms divided by height in meters squared.
Maternal BMI were categorized into three groups recommended by the Working Group on Obesity in China: BMI < 18.5 was defined as Underweight, BMI≥24.0 kg/m^2 was defined as overweigh & Obesity.

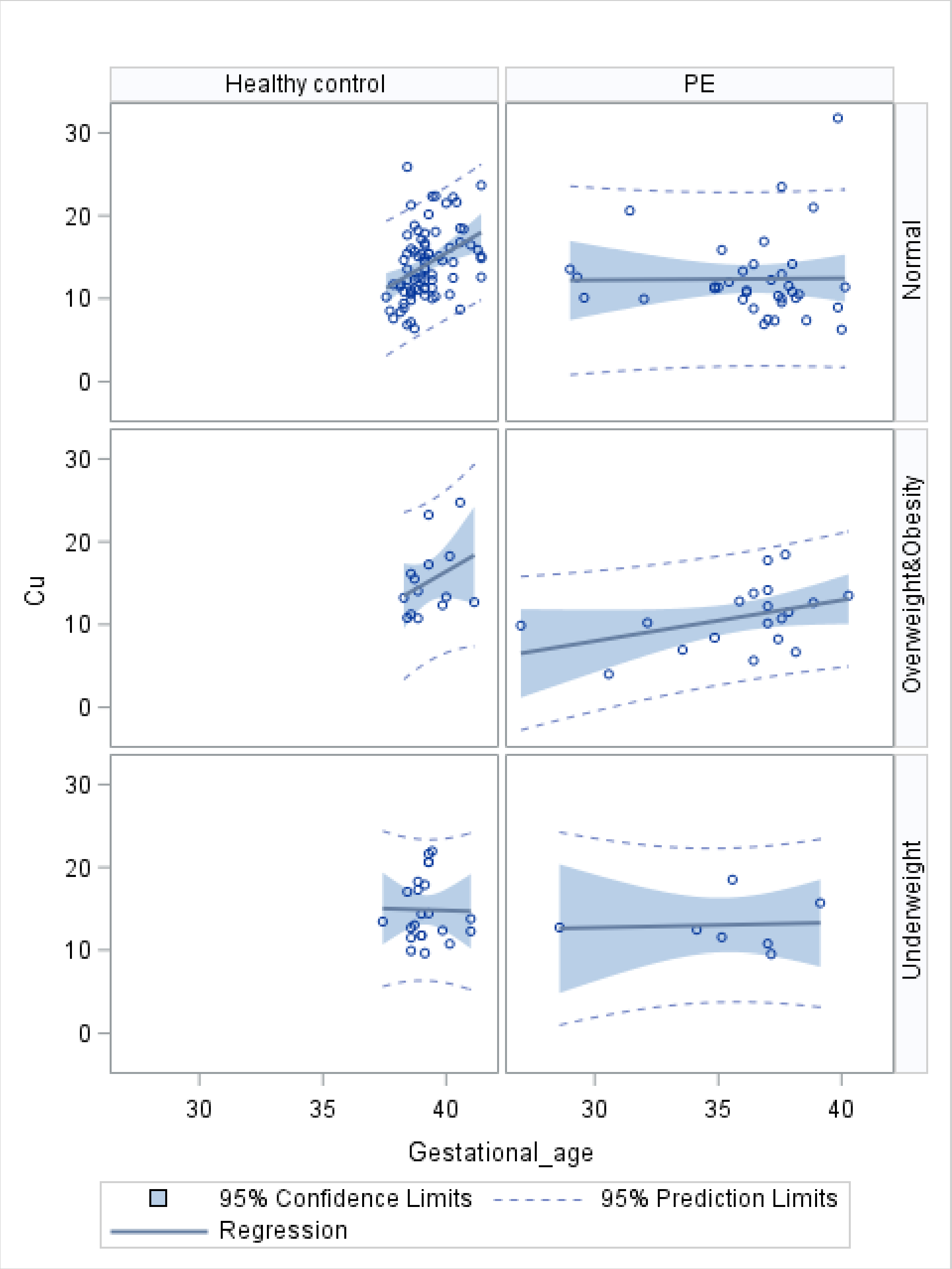

Figure S6. Placental Cu distribution across gestational age (weeks) in the healthy control group and the preeclampsia (PE) group, categorized by maternal pre-pregnancy body mass index (BMI) groups. BMI as weight in kilograms divided by height in meters squared.
Maternal BMI were categorized into three groups recommended by the Working Group on Obesity in China: BMI < 18.5 was defined as Underweight, BMI ≥ 24.0 kg/m^2 was defined as overweigh & Obesity.

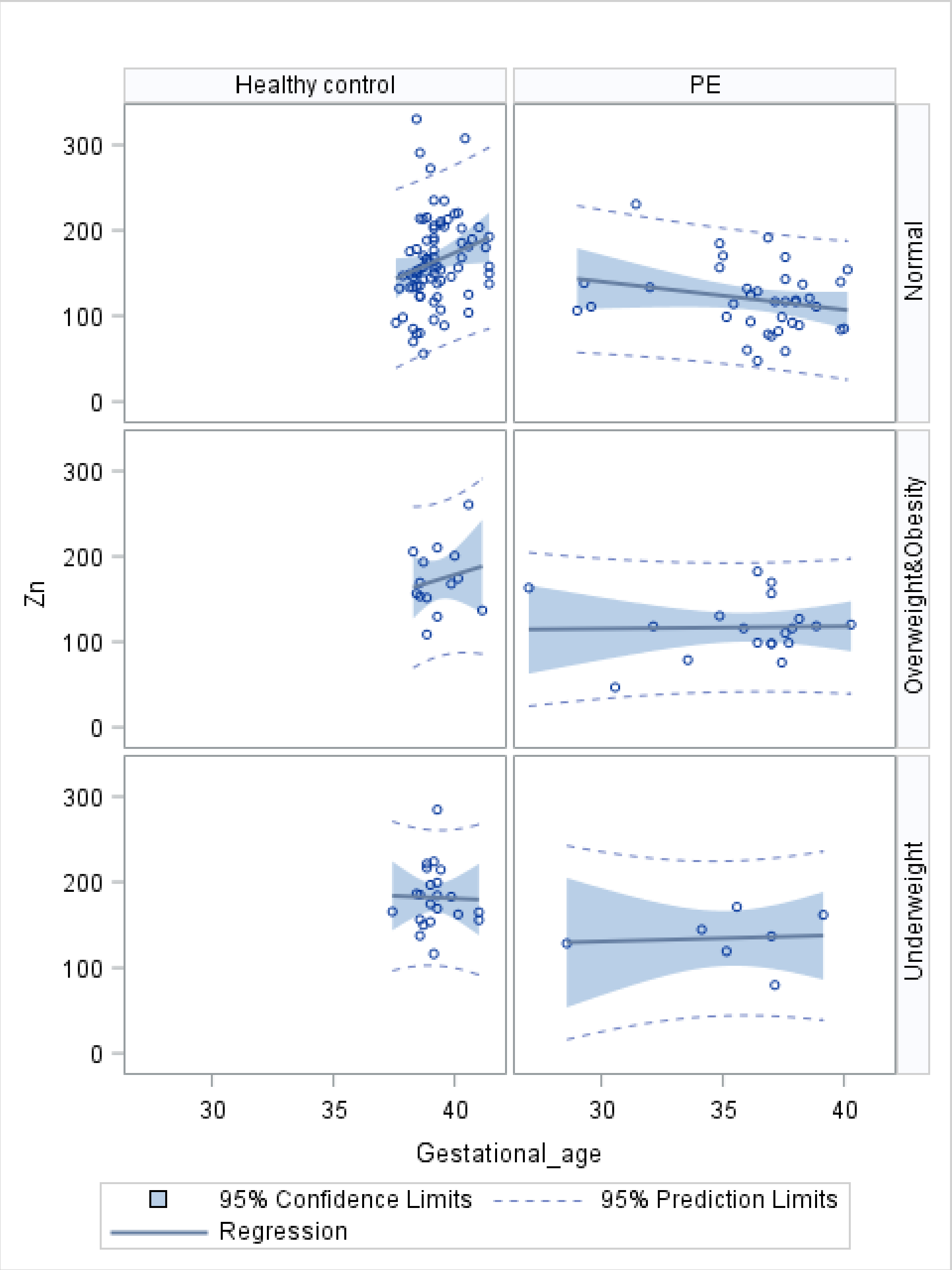

Figure S7. Placental Zn distribution across gestational age (weeks) in the healthy control group and the preeclampsia (PE) group, categorized by maternal pre-pregnancy body mass index (BMI) groups. BMI as weight in kilograms divided by height in meters squared.

Maternal BMI were categorized into three groups recommended by the Working Group on Obesity in China: BMI < 18.5 was defined as Underweight, BMI ≥ 24.0 kg/m² was defined as overweigh & Obesity.

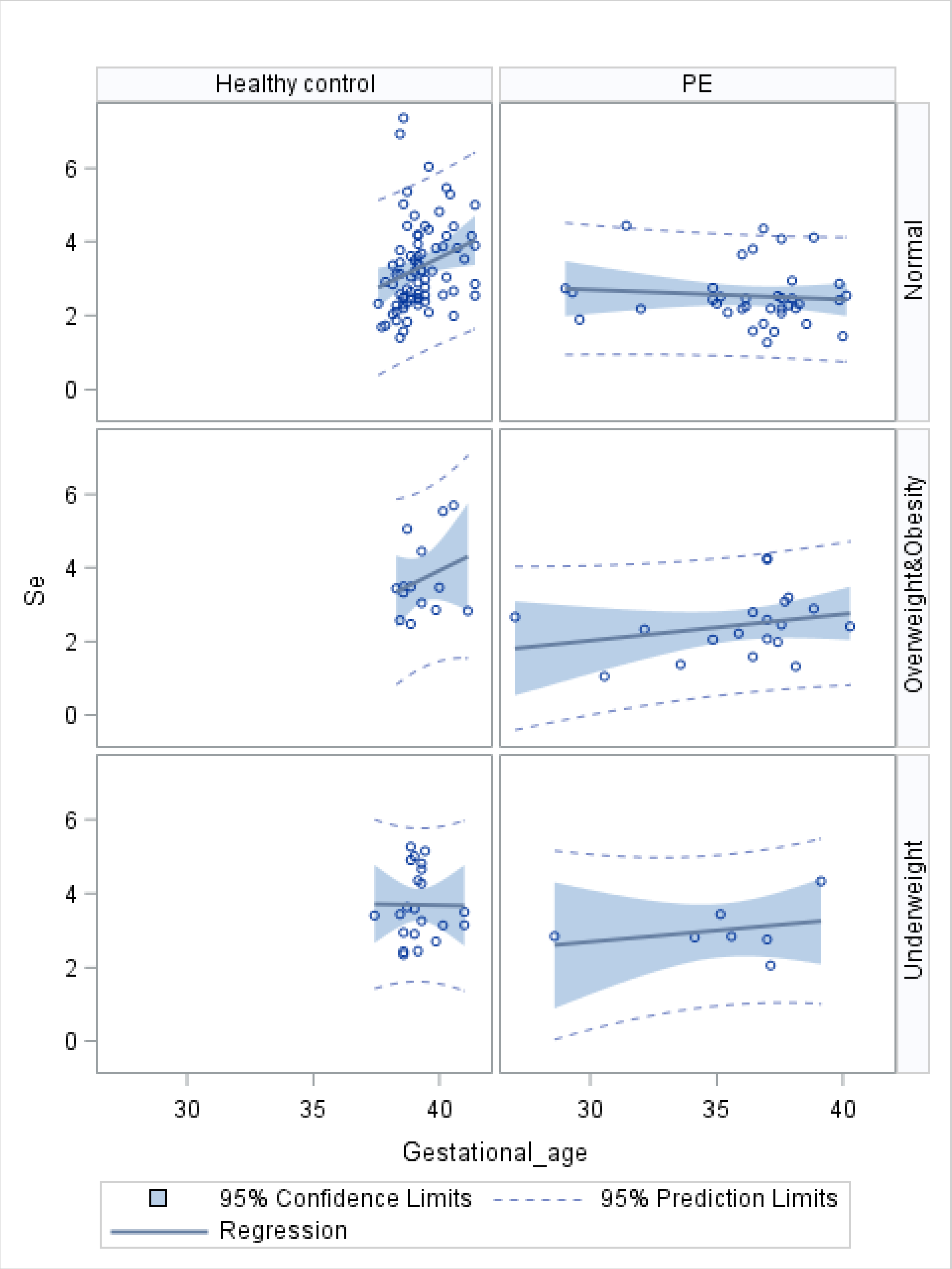

Figure S8. Placental Se distribution across gestational age (weeks) in the healthy control group and the preeclampsia (PE) group, categorized by maternal pre-pregnancy body mass index (BMI) groups. BMI as weight in kilograms divided by height in meters squared.

Maternal BMI were categorized into three groups recommended by the Working Group on Obesity in China: BMI < 18.5 was defined as Underweight, BMI ≥ 24.0 kg/m² was defined as overweigh & Obesity.

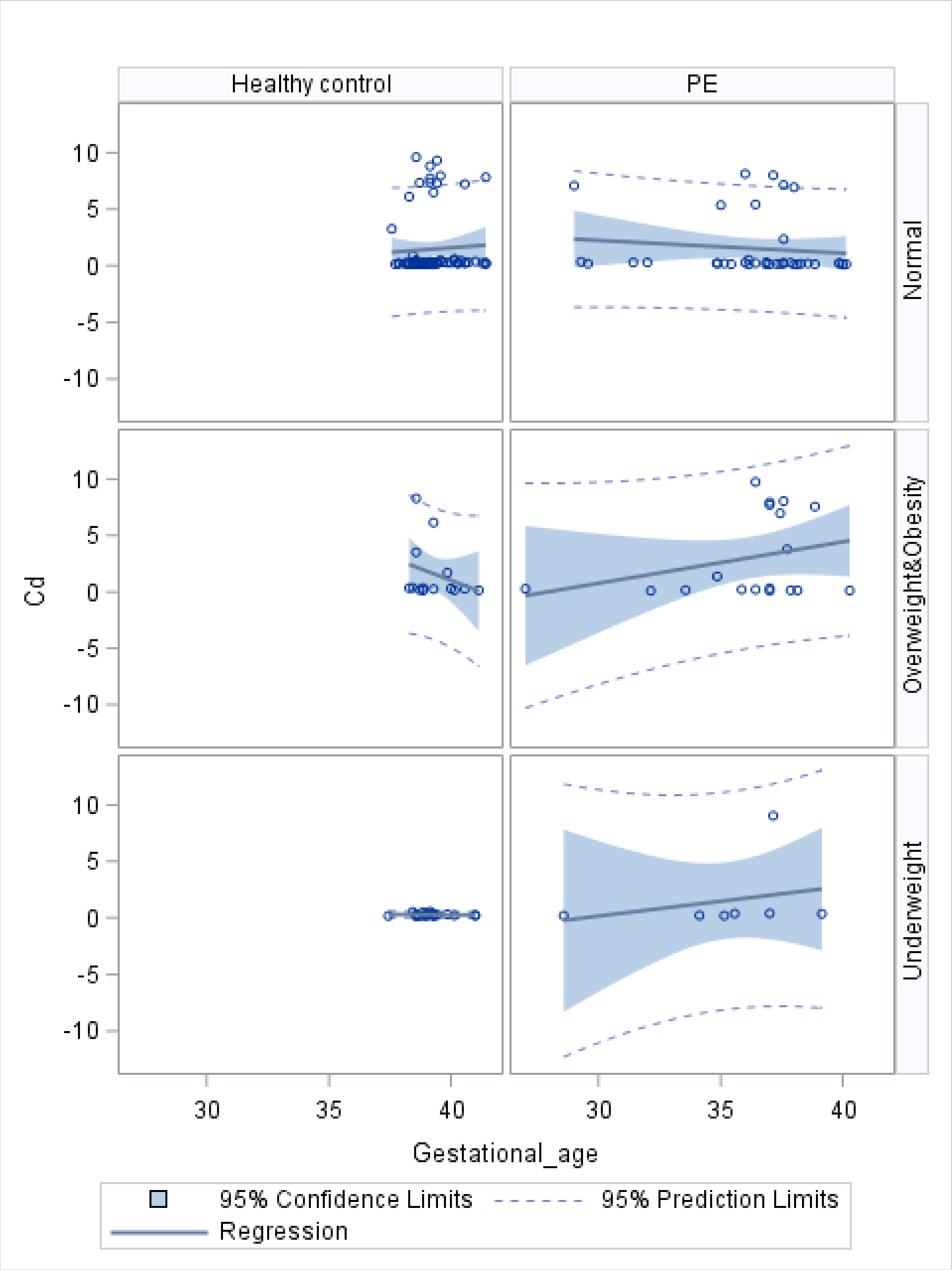

Figure S9. Placental Cd distribution across gestational age (weeks) in the healthy control group and the preeclampsia (PE) group, categorized by maternal pre-pregnancy body mass index (BMI) groups. BMI as weight in kilograms divided by height in meters squared.

Maternal BMI were categorized into three groups recommended by the Working Group on Obesity in China: BMI <18.5 was defined as Underweight, BMI≥24.0 kg/m^2 was defined as overweigh & Obesity.

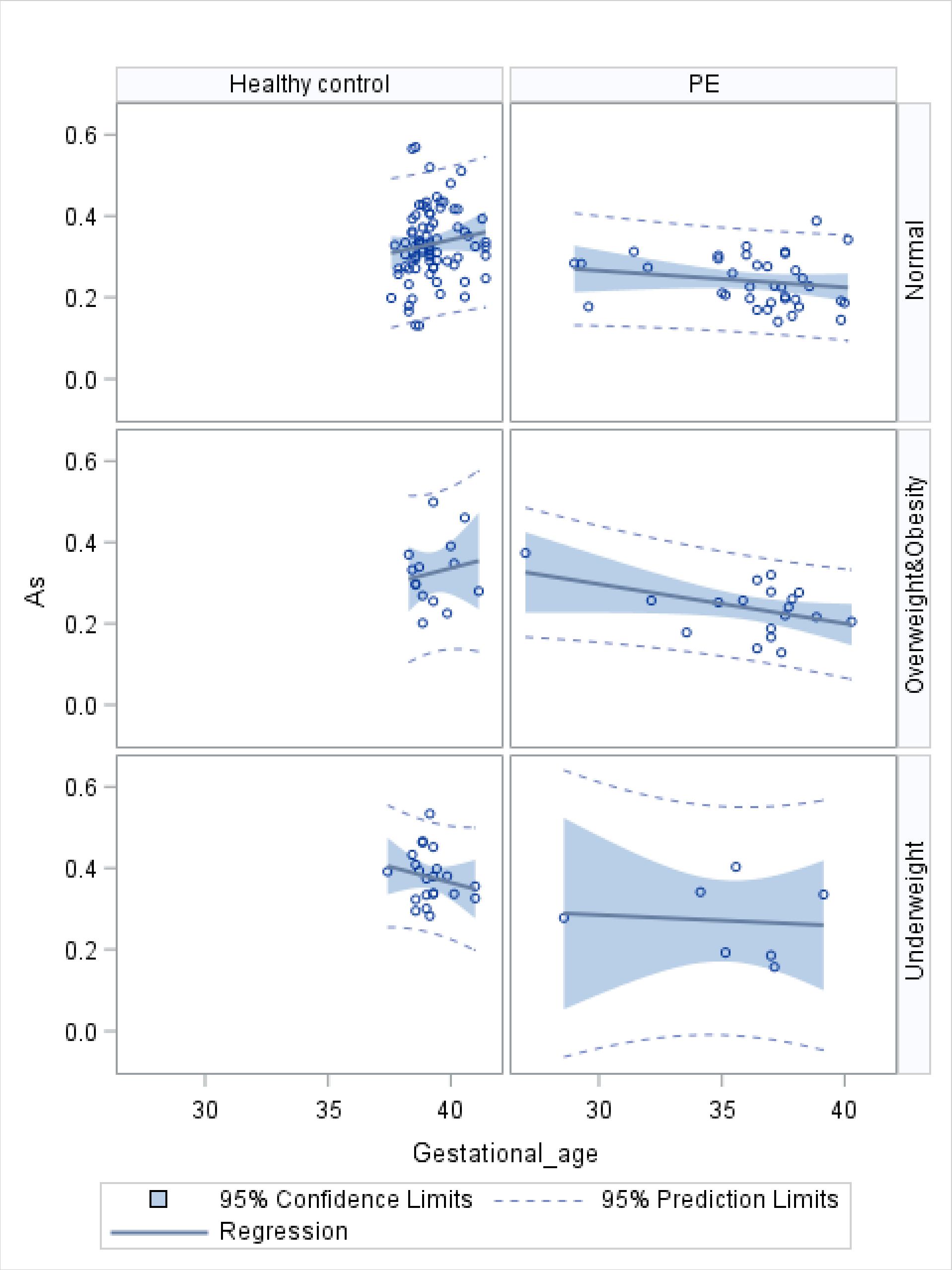

Figure S10. Placental As distribution across gestational age (weeks) in the healthy control group and the preeclampsia (PE) group, categorized by maternal pre-pregnancy body mass index (BMI) groups. BMI as weight in kilograms divided by height in meters squared.

Maternal BMI were categorized into three groups recommended by the Working Group on Obesity in China: BMI < 18.5 was defined as Underweight, BMI≥24.0 kg/m^2 was defined as overweigh & Obesity.

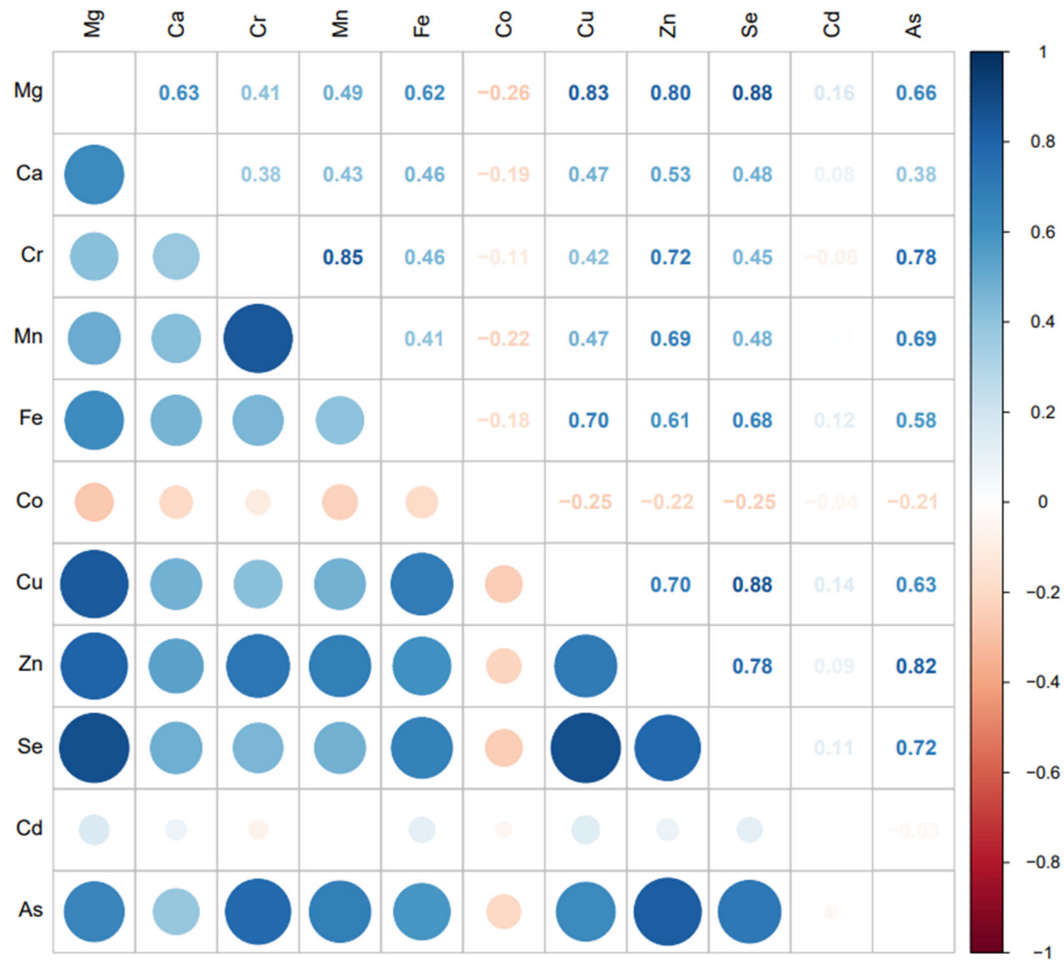

Figure S11. The correlation of metals/metalloids measured in placental for the whole study population (n = 176). Abbreviation: As: arsenic; Ca: calcium; Cd: cadmium; Co: cobalt; Cr: chromium; Cu: copper; Fe: iron; Mg: magnesium; Mn: manganese; Se: selenium; Zn: zinc

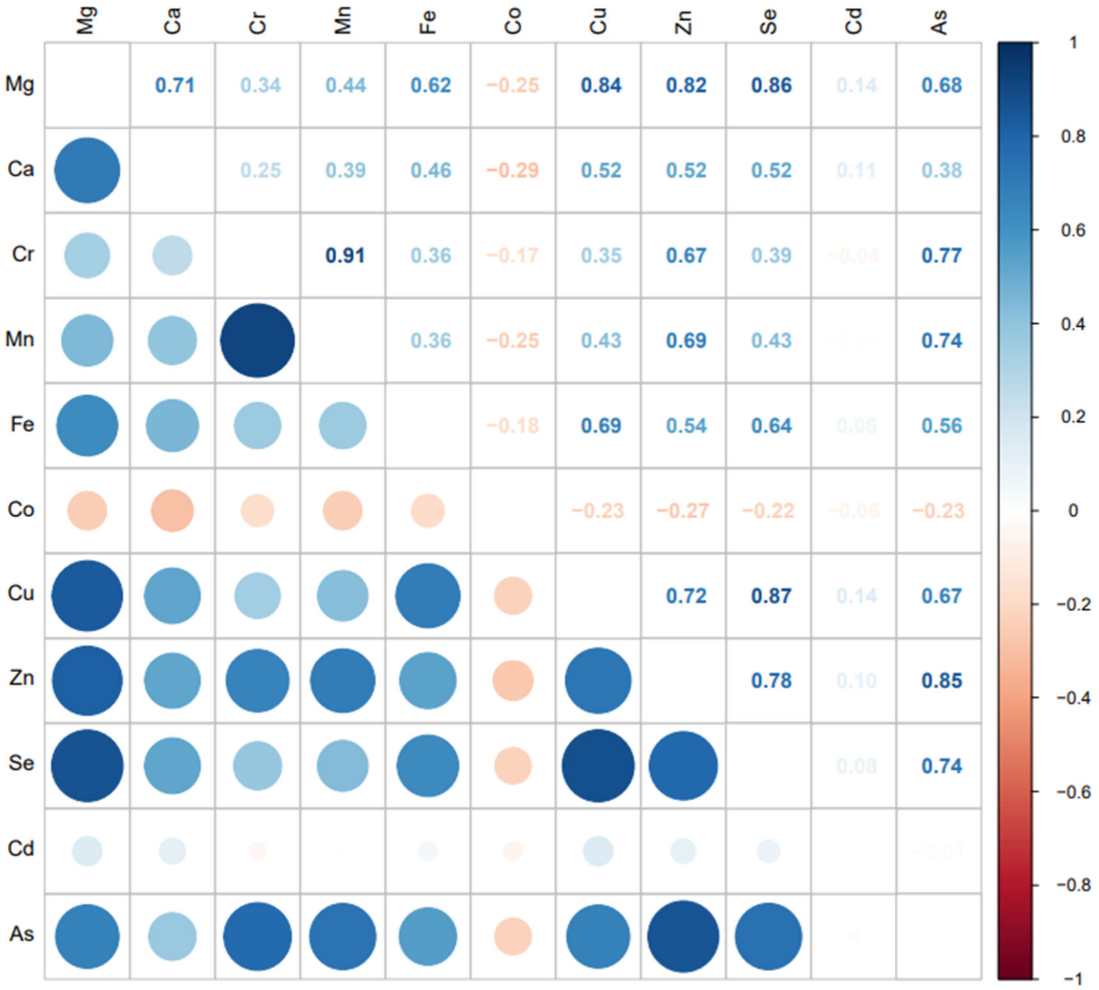

Figure S12. The correlation of metals/metalloids measured in placental for the healthy control subjects (n = 113). Abbreviation: As: arsenic; Ca: calcium; Cd: cadmium; Co: cobalt; Cr: chromium; Cu: copper; Fe: iron; Mg: magnesium; Mn: manganese; Se: selenium; Zn: zinc

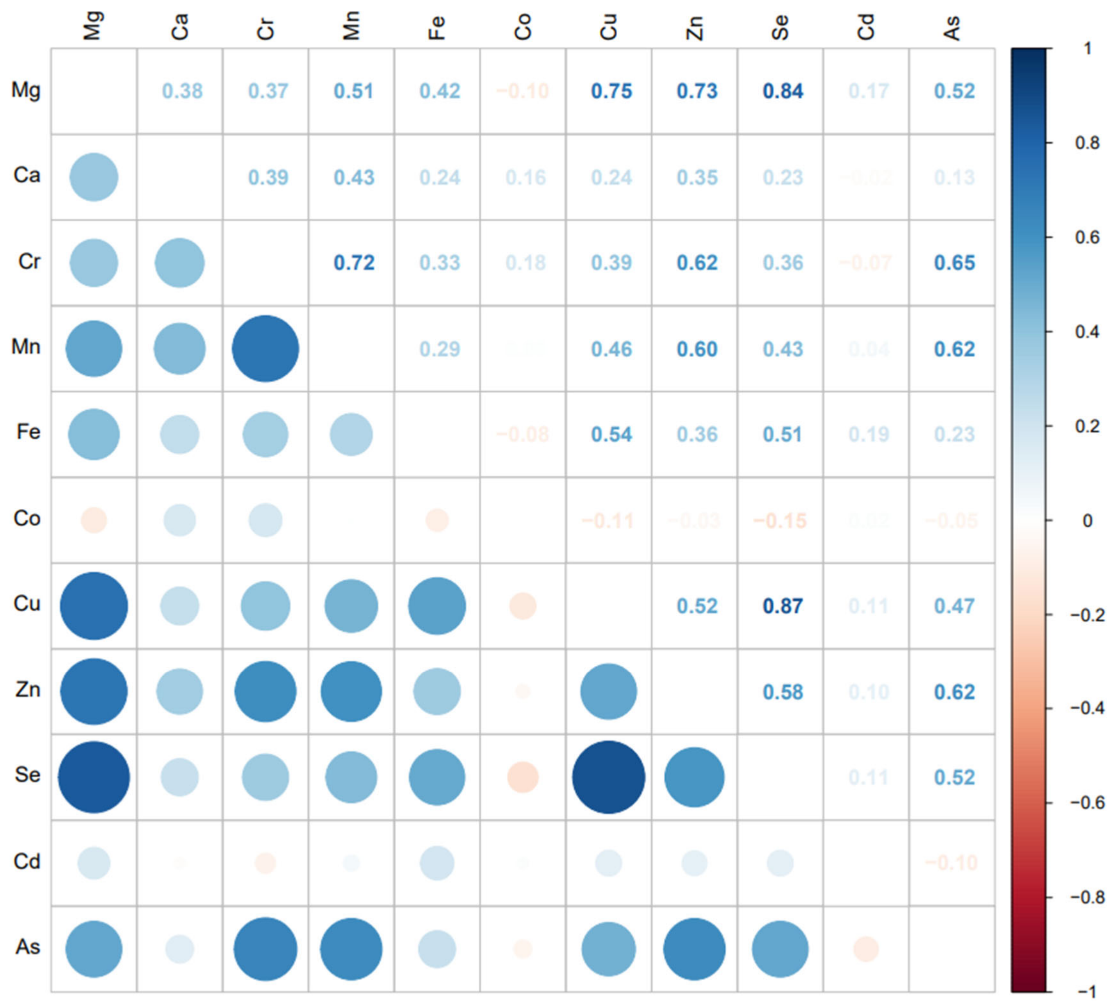

Figure S13. The correlation of metals/metalloids measured in placental for the preeclampsia group (n = 63). Abbreviation: As: arsenic; Ca: calcium; Cd: cadmium; Co: cobalt; Cr: chromium; Cu: copper; Fe: iron; Mg: magnesium; Mn: manganese; Se: selenium; Zn: zinc

Supplemented information on the assay of metal and metalloid in placenta

Placentas were collected promptly following delivery or termination of pregnancy, assigned codes, frozen, and stored in -80 °C freezers at the biobank of the Shanghai International Peace Maternity and Child Health Hospital (IPMCH) until analysis. Subsequently, all placental samples were sent in a batch to the assessment laboratory at Fudan University for determination using inductively coupled plasma mass spectrometry (ICP-MS, NexION 300X, PerkinElmer, USA).

1. Before measurement, placentas were defrosted at 4 °C and rinsed with deionized water to remove residual blood, and subsequently dried using filter paper.
2. Approximately 0.5 grams of wet weight was weighed on an analytical balance and placed in a polytetrafluoroethylene digestion vessel
3. 5 ml of concentrated nitric acid (70% ultrapure nitric acid from BASF, Germany) was added. Microwave digestion system (CEM MARS, USA) was performed until complete dissolution, the digestion system operate at a maximum power of 1600W.
4. Followed by diluting to 50ml with deionized water for analysis.

Protocol	Power (W)	Power enters (%)	Ramp (min)	Parameter (C°)	Holdtime (min)
1	1600	100	10	120	5
2	1600	100	8	190	20

Notes.

- In the test, all the standard substances used were purchased from PerkinElmer (standard solution, PerkinElmer mixed standard No. 3, 10 μg/mL, 5% nitric acid;)
- All reagent solutions were ultrapure, and water was produced by a purification system (Direct-Q-UV Millipore, Billerica, MA, USA).
- The initial calibration of ICP-MS and ICP-OES was verified by cesium (GSB 04-1745-2004).
- The operators were blind to the groups of samples.
